# Simultaneously Enhanced Permeability and Selectivity of Pebax-1074-Based Mixed-Matrix Membrane for CO_2_ Separation

**DOI:** 10.3390/membranes15010026

**Published:** 2025-01-13

**Authors:** Rujing Hou, Junwei Xie, Yawei Gu, Lei Wang, Yichang Pan

**Affiliations:** State Key Laboratory of Materials-Oriented Chemical Engineering, College of Chemical Engineering, Nanjing Tech University, Nanjing 210009, China; rujing.hou@njtech.edu.cn (R.H.); 202261104011@njtech.edu.cn (J.X.); 202362042052@njtech.edu.cn (Y.G.); 202362042066@njtech.edu.cn (L.W.)

**Keywords:** mixed-matrix membrane, metal–organic framework, carbon capture, grafting

## Abstract

Membrane technology is a promising methodology for carbon dioxide separation due to its benefit of a small carbon footprint. However, the trade-off relationship between gas permeability and selectivity is one obstacle to limiting its application. Herein, branched polyethyleneimine (BPEI) containing a rich amino group was successfully grafted on the surface of the metal–organic framework (MOF) of AIFFIVE-1-Ni (KAUST-8) through coordination between N in BPEI and open metal sites in the MOF and with the resultant maintained BET surface area and pore volume. Both the strengthened CO_2_ solubility coefficients coming from the additional CO_2_ adsorption sites of amino groups in BPEI and the reinforced CO_2_ diffusivity coefficients originating from the fast transport channels created by KAUST-8 led to the promising CO_2_ separation performance for KAUST-8@BPEI/Pebax-1074 MMM. With 5 wt.% KAUST-8@BPEI loading, the MMM showed the CO_2_ permeability of 156.5 Barrer and CO_2_/N_2_ selectivity of 16.1, while the KAUST-8-incorporated MMM (5 wt.% loading) only exhibited the CO_2_ permeability of 86.9 Barrer and CO_2_/N_2_ selectivity of 13.0. Such enhancement is superior to most of the reported Pebax-1074-based MMMs for CO_2_ separation indicating a wide application for the coordination method for MOF fillers with open metal sites.

## 1. Introduction

Urgent action is required to limit warming to 2 °C by the year 2100 according to Intergovernmental Panel on Climate Change (IPCC) [1,2]. One of the main greenhouse gases is carbon dioxide. Various techniques have been developed for carbon capture including distillation, sorption, and membrane separation. The mature market-dominated technology of distillation and sorption, such as liquid amine-scrubbing and sorbents adsorption, which have a large carbon footprint and equipment footprint for CO_2_ capture, due to the phase change involved in the distillation and the absorbent regeneration step in sorption [3,4]. Comparatively, membrane technology stands out due to its high energy efficiency, small physical footprint, and low carbon footprint [5,6]. Baker’s study demonstrated the potential of membranes for carbon capture by showing a reduction of 44% energy cost relative to the amine absorption method when increasing the CO_2_ feed content from 10% to 30% with CO_2_ purity more than 99%, which meets the requirement set by the Department of Energy [7].

With intensive studies of membrane separation, there is yet industrial application for flue gas treatment. For polymeric membranes, the main obstacle is the trade-off relationship between gas permeability and selectivity that the increased gas permeability is generally coupled with the reduced selectivity, and vice versa [8,9,10]. For inorganic membranes, the inevitable intercrystallite defects limit its further scale-up [5]. Correspondingly, the mixed-matrix membranes (MMMs) fabricated by incorporating nanoparticle filler as the dispersed phase into the continuous phase of polymer matrix have gained increasing attention in recent years because they combine the advantages of solvent-processability from polymers and precise pore-regulation ability from nanoparticles, which is helpful to break the trade-off issue [8]. Diverse particles have been investigated as fillers in the MMMs including inorganic metal oxide, organic porous aromatic framework (PAF) [11], and the organic–inorganic hybrid of mental-organic framework (MOF) [12].

Among these, metal–organic framework (MOF) is one of the most popular and efficient fillers due to its highly controllable pore size, shape, and functionality arising from the diversity of metal ions and ligands that can accommodate the specific application scenario [13,14]. For example, the 5 wt.% loading of micron-sized hollow ZIF-8 in the MMM made the CO_2_ permeability 224.6% and 63.5% higher than the neat PEO membrane and nanosized hollow ZIF-8/PEO MMM due to the accumulated transport highway constructed by the micron-sized hollow ZIF-8 [15]. The 5 wt.% loading of Cu@Al(bpydc) in Pebax accelerated the diffusion and solubility of CO_2_ in the MMMs and led to the simultaneous improvement of CO_2_ permeability and CO_2_/CH_4_ selectivity by 92.90% and 27.10%, respectively [16]. A similar concurrent enhancement for CO_2_ permeability and CO_2_/N_2_ selectivity was also reported in the 10 wt.% PHZ-2/Pebax MMMs owing to the improved free volume and low gas transfer resistance caused by the hollow structure of filler [17].

**Figure 1:**
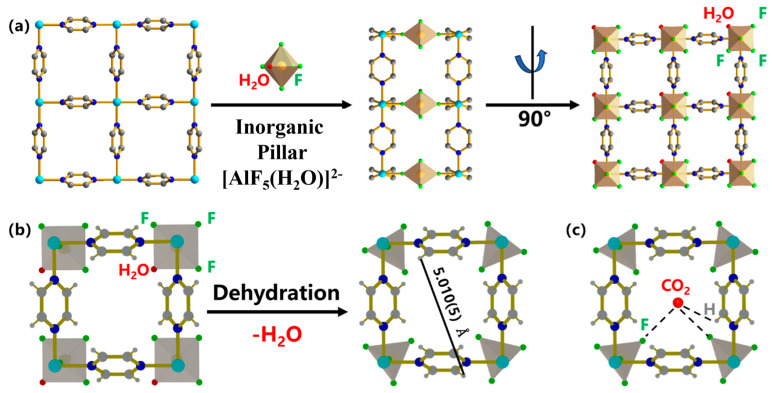
(**a**) Schematic diagram of KAUST-8 formation, (**b**) Dehydration process of KAUST-8, and (**c**) CO_2_ interaction sites with KAUST-8. (Ni, cyan; N, blue; C, grey; O, red; F, green; Al, orange).

As a pillar-layered metal–organic framework, AIFFIVE-1-Ni (KAUST-8) with periodic square-grid layer established by linking Ni(II) ion with pyrazine ligand and further pillared by the [AlF_5_(H_2_O)]^2−^ anion in the third dimension (primitive cubic (pcu) topology) was applied as fillers in this study (Figure 1a) [18,19,20,21]. The pillar was demonstrated as an octahedral with an Al^3+^ metal center accommodated with five F atoms and one H_2_O molecule with three F atoms and one H_2_O molecule facing the 1D channel (Figure 1b, left). Upon dehydration, with one coordinated H_2_O molecule lost away from the Al^3+^ site, the pillar geometry changed from a square to a trigonal bipyramidal-like structure with a reported pore size of around 5 Å (Figure 1b). The reported structure demonstrated double loaded site for CO_2_ with both electropositive interaction between C with F atoms in pillars (F···C_CO2_ = ~2.8 Å) and electronegative O with H in pyrazine ligand (H···O_CO2_ = ~2.6 Å, Figure 1c) [13,21,22]. The open metal site of Al^3+^ was declared as a barrier for more CO_2_ loading as it blocks its simultaneous interaction with the four F atoms [14,21]. Meanwhile, the open metal site was well known for anchoring the desired functional moiety to boost the targeted application [23,24,25]. To utilize the CO_2_ affinity sites and further enhance their CO_2_ separation ability, branched polyethyleneimine (BPEI)-grafted KAUST-8 was constructed and applied as fillers in Pebax 1074. Pebax 1074 was chosen as the polymer matrix because it consists of 55% soft phase polyethylene oxide and 45% hard phase polyamide 12, which can correspondingly supply the CO_2_ affinity property and the mechanical strength [26]. With 5 wt.% SAPO zeolite, the Pebax-1074-based MMM exhibited the CO_2_ permeability of 98.2 Barrer and CO_2_/N_2_ selectivity of 72 [27]; The 8 wt.% loading of ZnO in Pebax 1074 accelerated the CO_2_ permeability and CO_2_/N_2_ selectivity of the MMM from 110.67 to 152.27 Barrer and 50.08 to 62.15, separately [28]. The function of the grafted BPEI in KAUST-8 and its mechanism for CO_2_ separation were thoroughly investigated in this work.

## 2. Experimental Section

### 2.1. Materials

Nickel (II) acetate (Ni(CH_3_COO)_2_, 99%), pyrazine (C_4_H_4_N_2_, 99%), hydrofluoric acid (HF, 40 wt.% in H_2_O), and n-butanol (C_4_H_10_O, 99%) were purchased from Shanghai Aladdin Biochemical Technology Co., Ltd., Shanghai, China. Aluminum (III) hydroxide (Al(OH)_3_, AR), methanol (CH_4_O, 99.9%), and ethanol (C_2_H_6_O, 99.9%) were obtained from Sinopharm Chemical Reagent Co., Ltd., Shanghai, China. Branched polyethyleneimine (BPEI, 99%, M_n_ = 600 g⋅mol^−1^) was purchased from Merck Ltd., Beijing, China. Pebax 1074 polymer pellet was provided by Arkema Inc., Beijing, China. Gases of N_2_, CO_2_, and He were supplied by Nanjing Chuangda Group Co., Ltd., Nanjing, China. All purchased materials were used as received without further purification.

### 2.2. Preparation of KAUST-8

The KAUST-8 was synthesized according to our previous work [29]. Pyrazine (1.80 g) and nickel acetate (2.20 g) were separately dissolved in ethanol (6 mL) and deionized water (3 mL), followed by mixing and sonicating for 3 min. Aluminum hydroxide (0.7 g) was dissolved in a mixed solvent of HF and deionized water (7 mL, *v*:*v* = 6:1). The above two solutions were then mixed and sonicated for 3 min. The mixture was poured into a 50 mL Teflon liner and heated to 80 °C for 6 h. After cooling down to room temperature, the sky-blue product (Figure S1a) was collected by centrifugation and washed with deionized water three times and ethanol once. Finally, the product was dried at 60 °C for 24 h.

### 2.3. Preparation of KAUST-8@BPEI

The BPEI-grafted KAUST-8 was synthesized according to the reported work (abbreviated as KAUST-8@BPEI) [25]. BPEI (0.3 g) was dissolved in methanol (20 mL) and sonicated for 10 min. KAUST-8 (2.0 g) was added to the above solution and followed by stirring at 25 °C for 24 h. Then, KAUST-8@BPEI (Figure S1b) was collected by washing with methanol three times. Finally, the product was dried at 70 °C for 24 h.

### 2.4. Membranes Fabrication

The membranes were synthesized according to our previous work [30]. Pebax 1074 was dried in a vacuum oven at 80 °C for 6 h before use to remove any adsorbed moisture. The ratio of polymer/(polymer + solvent) is controlled at 2.5 wt.%. For the neat membrane fabrication, Pebax 1074 was dissolved in n-butanol at 90 °C under a water bath for 3 h. After dissolution, the solution was poured rapidly into the Teflon dish followed by placing in an oven at 60 °C for 14 h to obtain the membrane. Finally, the membrane was carefully removed from the Teflon dish and dried in a vacuum oven at 60 °C for 12 h to remove any residual solvent before the gas permeation test. The fabrication of the MMMs was similar to the neat membrane. The quantitative MOF (KAUST-8 and KAUST-8@BPEI) was dispersed in n-butanol and followed by stirring for 3 h. At the same time, Pebax 1074 was dissolved in n-butanol and heated for 3 h. Then, the Pebax 1074 solution was gradually added to the MOF-dispersed solution in 2 steps (1/10 for the first time and 9/10 for the second time) and each time it was further stirred at 90 °C for 2 h. The following procedures were conducted in the same way as the neat membrane. For easy note, the KAUST-8 and KAUST-8@BPEI-based MMMs were separately labeled as KAUST-8/Pebax-X and KAUST-8@BPEI/Pebax-X, where X means the weight percentage of the loading (X = 2.5 wt.%, 5 wt.%, and 10 wt.%). Other loadings of more than 10 wt.% led to membrane cracks or serious agglomeration for the incorporated fillers. The thickness of all membranes was measured with a digital micrometer (Mitutoyo, Kanagawa, Japan) and around 60 ± 10 μm. The gas permeability and CO_2_/N_2_ selectivity of KAUST-8@BPEI/Pebax-5 with different thickness are shown in Table S1. It could be seen that membrane thickness has a slight effect on membrane separation performance.

### 2.5. Gas Permeation Measurements

**Figure 2:**
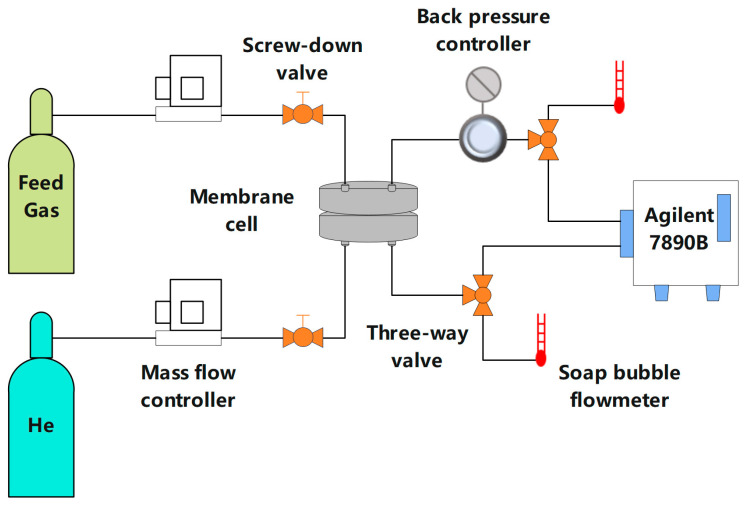
Schematic diagram for gas permeation test.

The membrane gas permeation measurements were carried out by using a home-made Wicke–Kallenbach technique shown in Figure 2. All gas permeation tests were carried out under the condition that the temperature was maintained at 25 °C and the feed pressure was controlled from 1 to 6 bar. To avoid gas leakage, the membrane sample with an effective membrane area (3.8 cm^2^) was placed in the sample holder and sealed with a rubber O-ring. The CO_2_/N_2_ feed gas mixture (*v*:*v* = 15:85) was fed to the membrane module. As shown in Table S2, the flow rate of 20 mL/min is the optimal experimental condition. Hence, the total flow rate of the feed side was controlled at 20 mL/min. The permeated gas was introduced into the gas chromatography (Agilent 7890, Santa Clara, CA, USA) and swept by Helium at a flow rate of 20 mL/min. After the test system was stable, the gas permeation data were tested three times and averaged. The gas permeability was calculated according to Equation (1).

(1) $$P_{i}=\frac{LN_{i}}{A\Delta P_{i}}$$

where *P_i_* is the gas permeability of the gas *i* (1 Barrer = 10^−10^ cm^3^(STP)∙cm∙cm^−2^∙s^−1^∙cmHg^−1^), *L* is the membrane thickness (cm), *N_i_* is the volumetric flux through the membrane of the gas *i* (cm^3^/s), *A* is the membrane effective area (cm^2^), and Δ*P_i_* is the pressure difference across the membrane (cmHg).

The selectivity (*α_i_*_/*j*_) of membrane was calculated according to Equation (2).

(2) $$\alpha_{i/j}=\frac{P_{i}}{P_{j}}$$

where *P_i_* and *P_j_* are the gas permeability of the gas *i* and *j*, respectively.

Based on the results obtained from the gas (CO_2_ and N_2_) adsorption, the solution coefficient (*S*) can be calculated according to Equation (3).

(3) $$S=\frac{c}{f}$$

where *S* is the solution coefficient (cm^3^(STP)∙cm^−3^∙cmHg^−1^), *c* is the concentration for adsorbed CO_2_ of the sample (cm^3^(STP)∙cm^−3^), and *f* is the corresponding upstream fugacity driving force (cmHg).

The diffusion coefficient (*D*) can be obtained indirectly based on the Equation (4).

(4) $$D=\frac{P}{S}$$

where *D* is the diffusion coefficient (cm^2^∙s^−1^).

### 2.6. Characterizations

X-ray diffraction (XRD) patterns of KAUST-8, KAUST-8@BPEI, and the corresponding MMMs were collected from the Rigaku Smartlab TM 9 KW equipment, Tokyo, Japan (Cu Kα radiation, 40 kV, 40 mA, and λ = 1.542 Å). Thermogravimetric analysis (TGA) of KAUST-8, KAUST-8@BPEI, and the corresponding MMMs was obtained from the thermal analysis system (NETZSCH STA 449, Selby, Germany) under N_2_ atmosphere from 30 °C to 800 °C with a heating rate of 10 °C/min. The morphologies of KAUST-8, KAUST-8@BPEI, and the corresponding MMMs were collected from field emission scanning electron microscopes (FESEM, S4800, Hitachi, Tokyo, Japan). X-ray photoelectron spectroscopy (XPS) of KAUST-8 and KAUST-8@BPEI was conducted on Kratos Amicus. The XPS spectra were conducted for C, N, Al, and Ni atoms and referenced to the C 1s level to correct the peak shift. The CO_2_ and N_2_ adsorption isotherms of the samples were measured by BSD-660M at 298 K and 77 K, respectively. Based on the N_2_ adsorption data, the multipoint BET (Brunauer–Emmett–Teller, Beijing, China) was used to determine the specific surface areas and pore widths of KAUST-8 and KAUST-8@BPEI. Fourier transform infrared (FTIR) spectroscopy analysis of the samples was performed on the Nicolet IS10 equipment (Waltham, MA, USA) in the wavenumber range of 400–4000 cm^−1^.

## 3. Results and Discussion

### 3.1. Characterization of KAUST-8@BPEI

**Figure 3:**
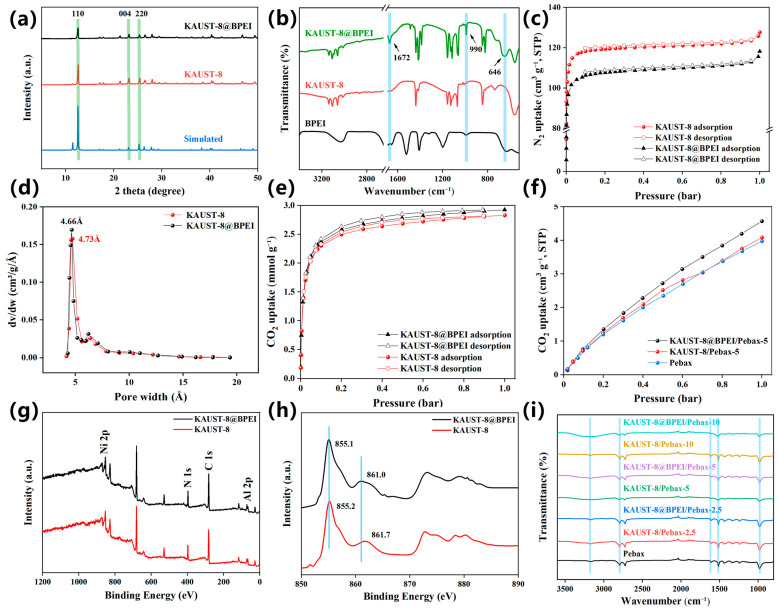
(**a**) XRD for KAUST-8 and KAUST-8@BPEI, (**b**) FTIR spectra for KAUST-8, KAUST-8@BPEI and BPEI, (**c**) N_2_ adsorption and desorption isotherms for KAUST-8 and KAUST-8@BPEI at 77 K, (**d**) Pore size distribution of KAUST-8 and KAUST-8@BPEI calculated from 77 K N_2_ adsorption isotherm based on the Horvath–Kawazoe model, (**e**) CO_2_ sorption isotherms for KAUST-8 and KAUST-8@BPEI at 298 K, (**f**) CO_2_ sorption isotherms for neat membrane, KAUST-8/Pebax-5 and KAUST-8@BPEI/Pebax-5 at 298 K, (**g**) XPS spectra of KAUST-8 and KAUST-8@BPEI, (**h**) Ni XPS spectra of KAUST-8 and KAUST-8@BPEI, and (**i**) FTIR spectra for membranes. All green and blue vertical lines are drawn to guide the eye.

The crystallinity of the synthesized MOF was examined by XRD patterns in Figure 3a. Both KAUST-8 and KAUST-8@BPEI exhibited the identical pattern relative to the simulated pattern and the prior reported work [21,29] with the main peaks of 110, 004, and 220 at 2θ of 12.96°, 23.34° and 25.74°, respectively. This indicates the right product and the BPEI modification did not affect the crystallinity of KAUST-8. The weaker intensity of the KAUST-8@BPEI relative to the pristine KAUST-8 might be due to the interaction between KAUST-8 and BPEI, which was consistent with the trend of the BPEI-modified UiO-66 [31]. The decomposition temperature (Figure S2) was up to 350 °C aligning with the previous work, which proved the favorable thermal stability and the potential application in industrial scale of KAUST-8 and KAUST-8@BPEI [32]. The weight loss before 200 °C was due to the loss of the coordinated water in the framework of KAUST-8 as shown in Figure 1b [21]. Compared to KAUST-8, the more reduced weight loss (~5%) after 800 °C for KAUST-8@BPEI was due to the grafted BPEI functional groups, which supported the existence of BPEI on KAUST-8@BPEI surface. Similar thermal weight loss phenomena were found for BPIE-grafted ZIF-8 and carbon nanotubes in the previous works [25,33]. The grating of BPEI on KAUST-8 was proved by the FTIR spectra (Figure 3b). Newly emerged peaks at wavenumber of 1672 cm^−1^ assigned to the bending vibration of NH and 990 cm^−1^ and 646 cm^−1^ assigned to the out-of-plan wagging vibration of NH were detected for KAUST-8@BPEI compared to the pristine KAUST-8, which confirmed the successful grafting of the BPEI on KAUST-8 particles [33] and was echoed by the weaker intensity in the XRD pattern (Figure 3a). The minor reduced BET surface area (Figure 3c, Table S3, 489.9 m^2^/g vs. 433.1 m^2^/g), pore volume (7.6% drop, Table S3), and the slightly narrowed pore size (Figure 3d, 4.73 Å vs. 4.66 Å) certified that one end of the BPEI was grated into KAUST-8 framework. The CO_2_ uptake of KAUST-8 was 2.83 mmol/g, which was consistent with Eddaoudi’s work [21]. Meanwhile, the enhanced CO_2_ adsorption property (3.3% up, Figure 3e) revealed that the other end of BPEI functionalized as CO_2_ adsorption site. This was further confirmed by the XPS results, which showed a slight left shift to the lower binding energy for Ni 2p from 855.2 eV to 855.1 eV (Figure 3h) after KAUST-8 was grafted with BPEI, suggesting an enhancement of electron density in Ni [31]. The confirmation for the covalent attachment of BPEI with the KAUST-8 framework was supported by the detected increased ratio of N/Ni^2+^ (Table S4).

### 3.2. Characterization of MMMs

**Figure 4:**
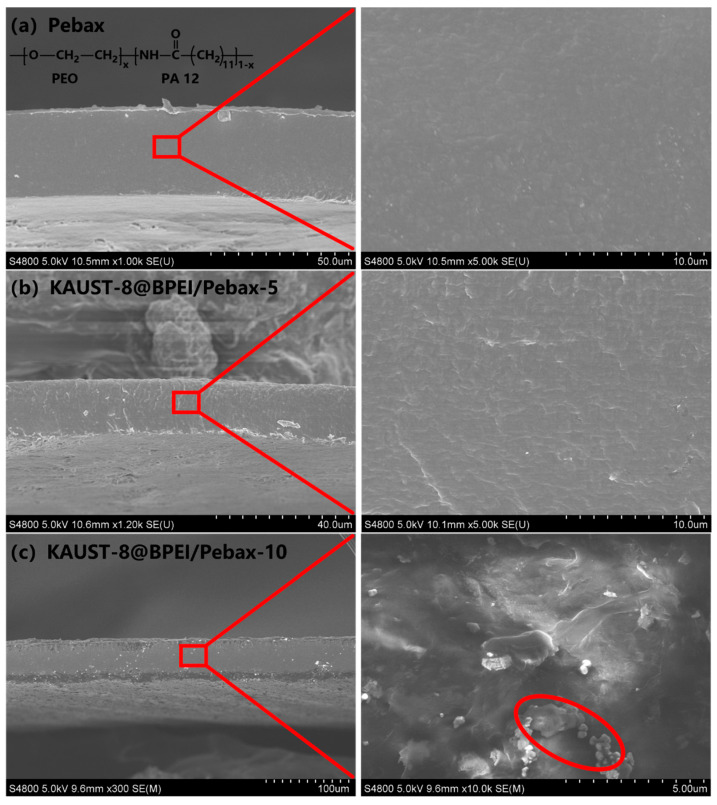
Cross-sectional SEM images for (**a**) neat membrane, (**b**) KAUST-8@BPEI/Pebax-5, and (**c**) KAUST-8@BPEI/Pebax-10, Left: low magnification, Right: high magnification, filler agglomeration highlighted in the red circle in the inset in Figure 4c.

The morphology of the neat membrane and MMMs was investigated by the SEM cross-sectional images (Figure 4a–c). The neat membrane demonstrated a smooth cross-sectional morphology (Figure 4a), which was consistent with our prior work [30]. With the addition of KAUST-8 and KAUST-8@BPEI at a loading lower than 10 wt.%, the MMMs showed a similar cross-sectional morphology to that of the neat membrane without obvious interface voids or even a visible interface between the polymer and the additive (Figure 4b and Figure S3a–c), reflecting the compatible interface and defect-free membrane structure. Such behavior matched our previous work [34]. The OH-PDCX/PIM-1 MMMs exhibited similar morphology to that of the neat PIM-1 membrane without obviously distinguishing between additive and polymer under the compatible solvent, leading to a more compatible polymer–additive interface and therefore to a more enhanced membrane performance relative to the obvious polymer–additive interface membrane sample (CO_2_/N_2_ enhancement: 22% vs. 4.4%). When additive loading up to 10 wt.%, no matter with KAUST-8 or KAUST-8@BPEI, an obvious additive agglomeration was observed (highlighted in the red circle in Figure 4c and Figure S3d). This is a general phenomenon that a high additive loading of more than 10 wt.% has the tendency to join together for stabilization due to the high surface energy of the particle [29,30]. The FTIR spectra of the neat membrane showed peaks at 981 cm^−1^, 1513 cm^−1^, 1608 cm^−1^, 2792 cm^−1^, and 3170 cm^−1^, which are assigned to the typical C–O–C, H–N–C=O, O–C=O, C–H and N–H groups in Pebax 1074 polymer (Figure 3i) [35,36,37]. With the incorporation of KAUST-8 or KAUST-8@BPEI from 2.5 wt.% to 5 wt.%, and 10 wt.%, typical peaks were retained, indicating there was no detectable chemical change through the FTIR examination and the existence of additive did not affect the crystallinity of the polymer matrix. For CO_2_ adsorption (Figure 3f), the KAUST-8 additive-incorporated KAUST-8/Pebax MMMs showed slightly higher CO_2_ adsorption property relative to the controlled sample (4.08 cm^3^/g vs. 3.98 cm^3^/g). This was caused by the CO_2_ affinity sites in the framework of KAUST-8 (CO_2_ with both electropositive interaction between C with F atoms in pillars and electronegative O with H in pyrazine ligand, Figure 1c) [13,21,22]. The enhancement degree was small due to the low loading of KAUST-8 (5 wt.%) in the polymer matrix. With BPEI further grafted on KAUST-8, an obvious improvement of CO_2_ adsorption to 4.57 cm^3^/g was detected, revealing the successful grating of BPEI in KAUST-8 framework and consistency to the result in XPS (Figure 3g,h), along with the reported enhanced CO_2_ adsorption for BPEI, which was grated into MOF as noted in Zheng’s work [25].

### 3.3. Separation Performance

#### 3.3.1. Loading Effect

**Figure 5:**
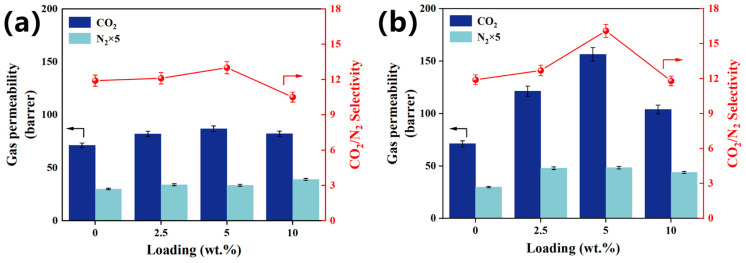
Loading effect on the change of gas permeability and CO_2_/N_2_ selectivity of (**a**) KAUST-8/Pebax MMMs and (**b**) KAUST-8@BPEI/Pebax MMMs. Arrows are drawn to guide the eye.

**Figure 6:**
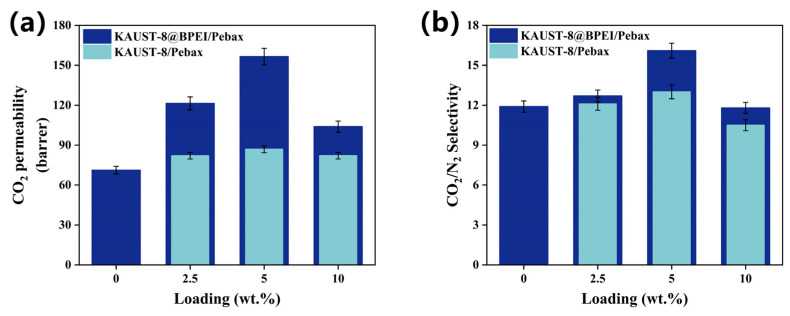
BPEI effect on the change of (**a**) CO_2_ permeability and (**b**) CO_2_/N_2_ selectivity of KAUST-8/Pebax MMMs and KAUST-8@BPEI/Pebax MMMs.

The loading effect was evaluated by increasing loading from 0 to 2.5 wt.%, 5.0 wt.%, and 10 wt.% in Figure 5. The neat membrane demonstrated CO_2_ permeability of 71.2 Barrer, N_2_ permeability of 6 Barrer, and ideal CO_2_/N_2_ selectivity of 11.9, which were within the range of the reported work since the CO_2_ permeability and CO_2_/N_2_ selectivity of the Pebax-1074-based MMMs, were influenced by several factors, such as Pebax-1074 from different batches, different kinds of solvent for membrane preparation, and so on. As a result, the membrane performance differences between the different Pebax-1074-based MMMs were significant [26,27,38,39]. With KAUST-8 loading (Figure 5a), CO_2_ permeability and CO_2_/N_2_ selectivity demonstrated a concomitant increasing trend till the optimal loading of 5 wt.% with an enhancement of 22% and 9%, respectively. Such an enhancement mainly came from the CO_2_ adsorption property of the embedded KAUST-8 additive (63 cm^3^/g in Figure 3e) as both F atoms in the pillar of [AlF_5_(H_2_O)]^2−^ and the H atoms in the pyrazine ligand can act as CO_2_ affinity sites. With further KAUST-8 loading to 10 wt.%, both CO_2_ permeability and CO_2_/N_2_ selectivity dropped, although they were still higher than the controlled sample. This was caused by the additive agglomeration at a higher loading, which reduced the ability of KAUST-8. A similar behavior was reported in the Y-abtc@Pebax MMMs for CO_2_/N_2_ separation [40]. By comparison, the KAUST-8@BPEI/Pebax MMMs demonstrated a similar gas permeation trend to that of the KAUST-8/Pebax MMMs under the function of various loading with the same optimal loading at 5 wt.%. However, higher loading also led to serious particle accumulation (Figure 4c). The difference for gas permeation change behavior under the function of KAUST-8@BPEI as the additive was explored in detail in Figure 6.

#### 3.3.2. BPEI Effect

In Figure 6, the KAUST-8@BPEI/Pebax MMMs showed a clear strengthened enhancement both for CO_2_ permeability (Figure 6a) and CO_2_/N_2_ selectivity (Figure 6b) relative to the KAUST-8/Pebax MMMs throughout various loading from 2.5 wt.% to 5 wt.%, and 10 wt.%. For example, at the optimal loading of 5 wt.%, the KAUST-8@BPEI/Pebax MMM showed a 120% increment for CO_2_ permeance and a 35.3% increment for CO_2_/N_2_ selectivity relative to the neat membrane, which were higher than most of the other Pebax-based MMM samples. Hence, the effect of the BPEI functionalization is effective and we intend to further enhance the role of BPEI in our future work. Generally, as an amine-functionalized moiety, BPEI possessed the ability for more CO_2_ adsorption. This was confirmed by the CO_2_ adsorption property both from the MOF particle (Figure 3e) and the corresponding membrane samples (Figure 3f), and it was consistent with prior work [25,31]. Consequently, a more CO_2_-selective and faster CO_2_ transport membrane structure was constructed. The reason for the specific function of BPEI for CO_2_ separation and the separation mechanism is discussed in the following section.

#### 3.3.3. Pressure, Temperature Effect and Long-Term Stability

**Figure 7:**
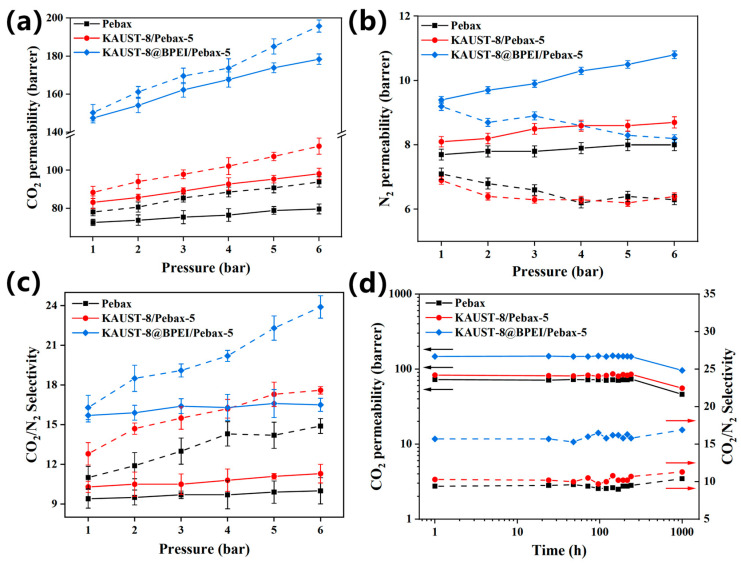
Pressure effect on the change of (**a**) CO_2_ permeability, (**b**) N_2_ permeability, and (**c**) CO_2_/N_2_ selectivity of neat membrane, KAUST-8/Pebax-5, and KAUST-8@BPEI/Pebax-5, dashed line: single gas condition, solid line: mixed gas condition. (**d**) Long-term gas permeation test of neat membrane, KAUST-8/Pebax-5, and KAUST-8@BPEI/Pebax-5 according to feed pressures of 1 bar, operated under 25 °C for mixed gas with a volume ratio of 15/85. Arrows are drawn to guide the eye.

Pressure effect on membrane separation performance was examined under both the single gas and mixed gas conditions (Figure 7). Generally, high pressure can lead to the following effect on membrane performance. Firstly, it can compact the membrane structure with the generated condensed fractional free volume, which corresponds to the reduced gas permeability; secondly, it can provide a more driving force across the membrane for the gas transportation and can be coupled with enhanced gas permeability; thirdly, the membrane can adsorb more condensable gas (such as CO_2_) and be plasticized with the resultant enlarged fractional free volume and the enhanced permeability. For CO_2_ permeability in the studied three samples (Figure 7a), they all increased with increasing the feed pressure under both single and mixed gas conditions and with a lower value under the mixture gas condition than that in the single gas condition. The increasing CO_2_ permeability with increasing feed pressure was caused by the plasticization effect. This was revealed by the opposite N_2_ permeability change trend under the single and mixed gas conditions (Figure 7b). Under the single gas condition, N_2_ permeability reduced gradually with the increasing feed pressure due to the polymer condensability effect under high pressure. However, under mixed gas conditions, N_2_ permeability gradually increased with increasing feed pressure, indicating that the membrane samples were already plasticized under the CO_2_/N_2_ mixture gases. Moreover, the presence of N_2_ in the mixed gas could prevent further adsorption and condensation of CO_2_ and reduce its solubility, which called for the competition sorption and was a general trend for mixed gas tests [26,28,41,42]. Hence, the increase rate for CO_2_ permeability under the mixed gas condition was lower than that of the single gas condition. Both enhanced CO_2_ and N_2_ permeability in the mixed gas condition also led to the fairly constant CO_2_/N_2_ selectivity relative to the single gas test (Figure 7c). Although it exhibited a plasticization effect in the MMM samples, the highest and constant CO_2_/N_2_ selectivity for the KAUST-8@BPEI/Pebax MMM in this study relative to the KAUST-8/Pebax MMM and the neat Pebax membrane (15.7 vs. 10.3 vs. 9.4) revealed the benefits of the synergistic function of KAUST-8 and BPEI grating for carbon capture.

Figure S4 showed the temperature effect on the change of gas permeability and CO_2_/N_2_ selectivity of KAUST-8@BPEI-5/Pebax. As the temperature rose, both CO_2_ and N_2_ permeability of KAUST-8@BPEI-5/Pebax increased because it facilitated the gas diffusion through the membrane. Similar to our previous work, due to the higher permeation activation energy, the N_2_ permeability was more susceptible to temperature, resulting in the larger degree of N_2_ permeability enhancement compared to the CO_2_ permeability and hence the reduced CO_2_/N_2_ selectivity [29,38]. Hence, a lower temperature is more suitable for separating CO_2_ and N_2_ with excellent selectivity. In addition, the three studied membrane samples demonstrated a similarly stable separation performance (Figure 7d) within the 240 h test under mixed gas condition, which demonstrated the highest CO_2_ permeability of 150.8 Barrer and the highest CO_2_/N_2_ selectivity of 16.5 for the KAUST-8@BPEI/Pebax MMM compared to the KAUST-8/Pebax MMM and the neat Pebax membrane. As the testing time reached 1000 h, the CO_2_ permeability of all test samples decreased, accompanied by an increase in CO_2_/N_2_ selectivity. This phenomenon was due to the decreased fractional free volume caused by the physical aging in the membranes [42]. Generally, the permeability of larger gas molecules declines more rapidly than that of smaller ones. Since the molecule size of N_2_ is larger than CO_2_, the degree of decrease in N_2_ permeability is severer than that of CO_2_, resulting in the slight increase in CO_2_/N_2_ selectivity.

### 3.4. Separation Mechanism

**Figure 8:**
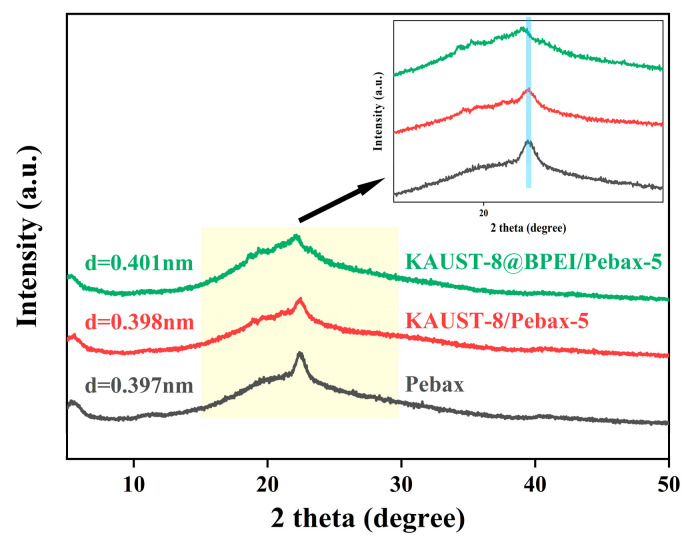
XRD for neat Pebax membrane, KAUST-8/Pebax-5, and KAUST-8@BPEI/Pebax-5 MMMs. The blue line is drawn to guide the eye.

**Figure 9:**
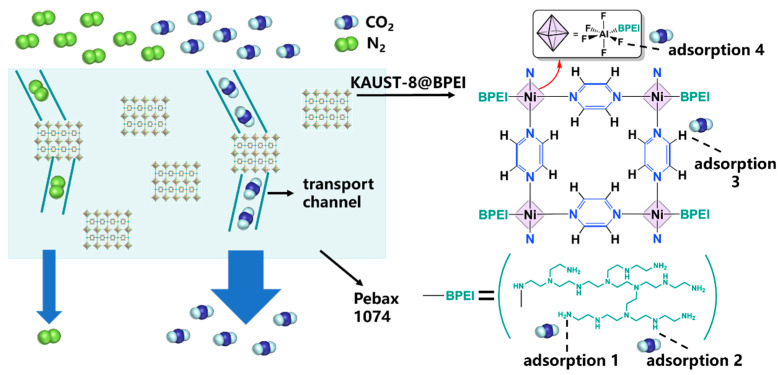
The gas transport behavior in KAUST@BPEI/Pebax MMM.

To clarify the function of KAUST-8 and BPEI for CO_2_ separation performance, the solubility and diffusivity coefficient deduced from the membrane gas sorption results (Figure 3f) are grouped in Table 1, and the XRD patterns for the d-spacing change are shown in Figure 8. Three membrane samples including neat membrane and with the optimal 5 wt.% loading MMMs with KAUST-8 and KAUST-8@BPEI were selected to clearly illustrate the effect of KAUST-8 and BPEI. As can be seen in Table 1, the neat Pebax membrane showed a CO_2_ solubility of 4.56 × 10^−2^ cm^3^(STP)∙cm^−3^∙cmHg^−1^ and CO_2_ diffusivity of 15.63 × 10^−8^ cm^2^∙s^−1^. With the addition of KAUST-8, the CO_2_ solubility was nearly retained with a slight enhancement of 2% but with an obvious CO_2_ diffusivity coefficient enhancement of 19%. The retained solubility coefficient was aligned to the nearly unchanged CO_2_ adsorption property by adding 5 wt.% into the polymer matrix, as illustrated in Figure 3f, and the obvious enhanced diffusivity coefficient was mainly from the porosity of the KAUST-8 framework with the reported pore size (~5 Å, Figure 1b). Similarly, the maintained N_2_ solubility was due to the lack of extra N_2_ affinity sites from the embedded KAUST-8 additives, and the minor increased N_2_ diffusivity coefficient was caused by the small addition of the porous structure of KAUST-8. Consequently, the obvious increment of CO_2_ diffusivity coefficient and the minor enhancement of N_2_ diffusivity coefficient led to the more boosted CO_2_ permeability than N_2_ permeability (Figure 5). With BPEI grafting, the amine group in BPEI contributed to the clearly strengthened CO_2_ solubility (15% up, Table 1) due to its CO_2_ affinity (Figure 3e,f) and slightly reduced N_2_ solubility due to the occupied surface area and pore volume in KAUST-8 through grating of BPEI (Figure 3c). Meanwhile, the branched structure of BPEI contributed to the further intensified CO_2_ and N_2_ diffusivity (Table 1) due to the fact that its branches can disrupt the polymer chain packing during the membrane formation process [25]. As a result, both CO_2_ and N_2_ permeability were further enhanced (Figure 6a). The d-spacing change in these three membrane samples (Figure 8) correlated to the diffusivity change in Table 1 and the corresponding membrane gas separation performance in Figure 5 and Figure 6. As seen in Figure 8, the XRD pattern for the neat Pebax membrane demonstrated a strong and typical diffraction peak at about 2θ of 22° aligned to the (001) form of the crystal from the PA diffraction [39]. The d-spacing for the neat membrane was 0.397 nm, which is similar to the reported work [39]. With the addition of KAUST-8, the typical peak at 2θ of 22° showed a slight left to 0.398 nm due to the disrupted polymer chain through the addition of KAUST-8 particle. With further BPEI grafting, the branched characteristic in BPEI intensified the polymer chain disruption and led to a reinforced left shift to 0.401 nm. This correlated to the enlarged d-spacing and extra gas transport channels for the continuously increased CO_2_ and N_2_ permeability (Figure 5 and Figure 6) and explained the enhanced gas diffusivity coefficient demonstrated in Table 1. Conclusively, the CO_2_ separation mechanism for the simultaneously enhanced gas permeability, and the selectivity is illustrated in Figure 9. In brief, the additional CO_2_ adsorption sites of -NH_2_ (adsorption site 1) and -NH- (adsorption site 2) from the grafted BPEI along with the H from pyridine (ligand, adsorption site 3) and F from pillar ([AlF_5_(H_2_O)]^2−^, adsorption site 4) contributed to the enhanced CO_2_ solubility. The porosity and branched structure of BPEI disrupted the polymer chain packing behavior and were responsible for the created fast gas transport channels as well as the increased CO_2_ and N_2_ diffusivity. The overall balanced function between the enhanced diffusivity coefficient for CO_2_ and N_2_ as well as the reduced N_2_ solubility coefficient led to the simultaneously enhanced CO_2_ permeability and CO_2_/N_2_ selectivity (Figure 6).

### 3.5. Comparison with Other Membrane Materials

**Figure 10:**
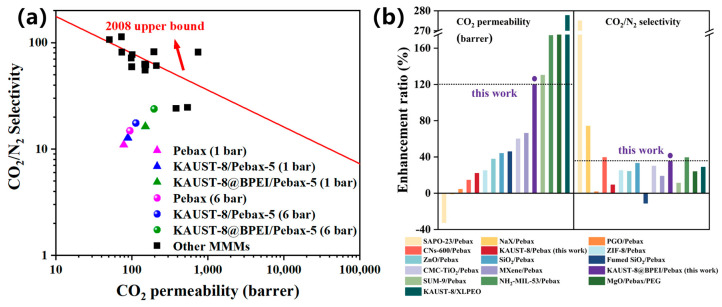
Membrane separation performance comparison with (**a**) 2008 Robeson upper bound and (**b**) enhancement ratio in terms of CO_2_ permeability and CO_2_/N_2_ selectivity (individual data refer to Table S5).

The CO_2_/N_2_ separation for the BPEI-grafted KAUST-8 MMMs was compared to other the Pebax-based MMMs by normalizing the enhancement of CO_2_ permeability and CO_2_/N_2_ selectivity to clearly demonstrate the function of the fillers. As shown in Figure 10a, even the gas separation performance of the representative MMM (KAUST-8@BPEI/Pebax-5) failed to surpass 2008 Robeson upper bound. It could be seen in Figure 10b that the proportion of improvement was relatively high [29,43,44,45]. The KAUST-8@BPEI/Pebax MMM showed 120% increment for CO_2_ permeance (left column) relative to the neat membrane, which was higher than most of the other MMM samples with the only exception of SUM-9, NH_2_-MIL-53, and MgO in the Pebax-based MMMs and the KAUST-8/XLPEO MMMs. Among these exceptions, all membrane samples showed a lower enhancement ratio for CO_2_/N_2_ selectivity (right column), with only one named NH_2_-MIL-53/Pebax exhibiting the simultaneously slightly higher CO_2_/N_2_ selectivity (39.4% vs. 35.3%) [45]. Such results confirmed the synergistic function of CO_2_-philic from BPEI and porous framework of KAUST-8 in Pebax for CO_2_/N_2_ separation. As a result, fillers in the MMMs specific for targeting in simultaneously increasing solubility and diffusivity coefficient for the targeted gases are a promising way to largely enhance the separation performance. In the long run, the grafting of BPEI can be applied to other kinds of MOFs, such as ZIF-8, UiO-66 and so on. Meanwhile, 6FDA-DAM or PIM-1 can be chosen as the polymer matrix to prepare the MMMs and achieve better performance.

## 4. Conclusions

In this work, branched polyethyleneimine (BPEI) was successfully grafted on the surface of KAUST-8 (AIFFIVE-1-Ni) through the coordination between the N element in BPEI and the open metal sites of Ni^2+^ in the framework of KAUST-8. The extra added CO_2_ adsorption sites from the amino group in the BPEI-grafted KAUST-8 combining the intrinsic CO_2_ adsorption sites of F from the inorganic pillar and H from the ligand led to the enhanced CO_2_ solubility coefficients in the KAUST-8@BPEI/Pebax MMMs. The additional fast transport channels arising from the porous structure of KAUST-8 and the enlarged d-spacing from the disrupted polymer chains caused by the branched structure of BPEI contributed to the overall enhanced gas diffusivity coefficient in the KAUST-8@BPEI/Pebax MMMs. The synergic strengthened CO_2_ solubility and diffusivity coefficients are responsible for the simultaneously enhanced CO_2_ permeability and CO_2_/N_2_ selectivity by the increment of 120% and 35%, respectively. The resultant MMMs exhibited the superior enhancement ratio for CO_2_/N_2_ separation and H_2_O/N_2_ separation performance to most of the reported membranes by demonstrating either higher gas permeability or selectivity or both higher than other membranes. The constant CO_2_/N_2_ selectivity in mixed gas under high pressure (6 bar) and stable long-term stability test running at 240 h for the KAUST-8@BPEI/Pebax MMMs relative to the counterpart samples highlights the efficiency of open metal sites engineering of MOF fillers in the MMMs for carbon capture. Moreover, the low cost of Pebax and filler provides the advantage for the industrial application of the MMMs.

## Figures and Tables

**Table 1:** Solubilities and diffusivities of CO_2_ and N_2_ for neat Pebax membrane, KAUST-8/Pebax-5, and KAUST-8@BPEI/Pebax-5 MMMs.

Membrane	CO_2_ Solubility [10^−2^ cm^3^(STP)∙cm^−3^∙cmHg^−1^]	CO_2_ Diffusivity [10^−8^ cm^2^∙s^−1^]	N_2_ Solubility [10^−2^ cm^3^(STP)∙cm^−3^∙cmHg^−1^]	N_2_ Diffusivity [10^−8^ cm^2^∙s^−1^]
Pebax	4.56	15.63	2.12	2.83
KAUST-8/Pebax-5	4.67	18.61	2.15	3.11
KAUST-8@BPEI/Pebax-5	5.23	29.92	1.58	6.14

## Data Availability

The original contributions presented in this study are included in the article/Supplementary Materials. Further inquiries can be directed to the corresponding author(s).

## Supplementary Materials

The following supporting information can be downloaded at: https://www.mdpi.com/article/10.3390/membranes15010026/s1, Figure S1: SEM images for (a) KAUST-8, Inset: KAUST-8 dispersion after soaking in n-butanol for 24 h and (b) KAUST-8@BPEI, Inset: KAUST-8@BPEI dispersion after soaking in n-butanol for 24 h; Figure S2: TGA for KAUST-8, KAUST-8@BPEI, neat membrane, KAUST-8/Pebax-5, and KAUST-8@BPEI/Pebax-5; Figure S3: Cross-sectional SEM images for (a) KAUST-8/Pebax-2.5, (b) KAUST-8@BPEI/Pebax-2.5, (c) KAUST-8/Pebax-5, and (d) KAUST-8/Pebax-10. Left: low magnification, Right: high magnification, filler agglomeration is highlighted in the red circle in the inset in Figure S3d; Figure S4: Temperature effect on the change of (a) CO_2_ and N_2_ permeability and (b) CO_2_/N_2_ selectivity of KAUST-8@BPEI-5/Pebax; Table S1: Membrane thickness effect on membrane separation performance of CO_2_ and N_2_ permeability and CO_2_/N_2_ selectivity; Table S2: Flow rates effect on membrane separation performance of CO_2_ and N_2_ permeability and CO_2_/N_2_ selectivity; Table S3: BET surface area and pore volume of KAUST-8 and KAUST-8@BPEI; Table S4: The atomic concentration of Al, C, N, and Ni for KAUST-8 and KAUST-8@BPEI from XPS, respectively; Table S5: Membrane separation performance comparison between this work and other published works. References [46,47,48,49,50,51,52,53] are cited in the Supplementary Materials.

## Abbreviations

The list of symbols and acronyms:

BPEI	Branched polyethyleneimine
D	Diffusion coefficient
FESEM	Field emission scanning electron microscopes
FTIR	Fourier transform infrared
HCP	Hyper-cross-linked polymer
HF	Hydrofluoric acid
IPCC	Intergovernmental Panel on Climate Change
KAUST-8	AIFFIVE-1-Ni
MMMs	Mixed-matrix membranes
MOF	Metal–organic framework
P	Permeability
PAF	Porous aromatic framework
PEO	Polyethylene oxide
PTMSP	Poly(1-trimethylsilyl1-propyne)
S	Solution coefficient
TGA	Thermogravimetric analysis
XLPEO	Crosslinked poly(ethylene oxide)
XPS	X-ray photoelectron spectroscopy
XRD	X-ray diffraction
