# Supplementary material for: Simultaneously Enhanced Permeability and Selectivity of Pebax-1074-Based Mixed-Matrix Membrane for CO2 Separation
Source: Membranes (Basel). 2025 Jan 13;15(1):26. doi: 10.3390/membranes15010026 (PMC11766677; doi:10.3390/membranes15010026)
Supplement: Supplementary file 1 [file membranes-15-00026-s001.zip › membranes-3380661-supplementary.pdf]

membranes

MDPI

Supplementary Materials

Article

Simultaneously Enhanced Permeability and Selectivity of Pebax-1074-Based Mixed-Matrix Membrane for CO_2 Separation

Rujing Hou, Junwei Xie, Yawei Gu, Lei Wang and Yichang Pan *

State Key Laboratory of Materials-Oriented Chemical Engineering, College of Chemical Engineering, Nanjing Tech University, Nanjing 210009, China; rujing.hou@njtech.edu.cn (R.H.); 202261104011@njtech.edu.cn (J.X.); 202362042052@njtech.edu.cn (Y.G.); 202362042066@njtech.edu.cn (L.W.)

* Correspondence: panyc@njtech.edu.cn

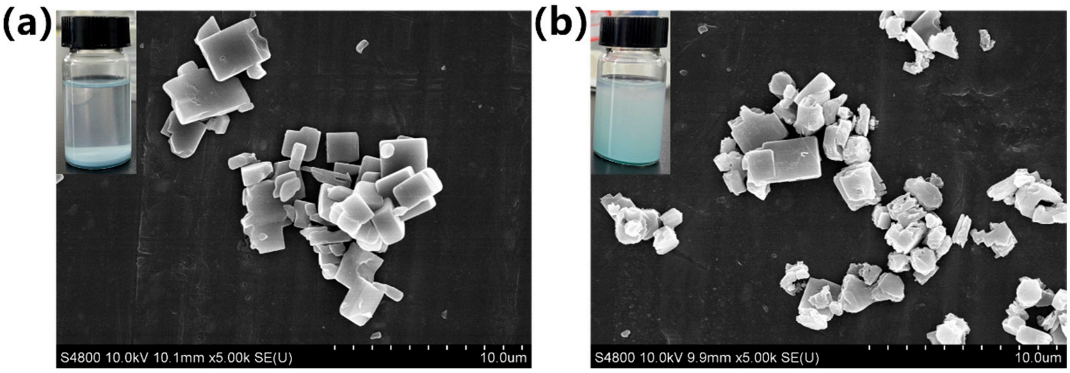

Figure S1. SEM images for (**a**) KAUST-8, Inset: KAUST-8 dispersion after soaking in n-butanol for 24 h and (**b**) KAUST-8@BPEI, Inset: KAUST-8@BPEI dispersion after soaking in n-butanol for 24 h.

The estimated particle size of KAUST-8 and KAUST-8@BPEI was 2 μm. The inhomogeneous dispersion of KAUST-8 in n-butanol was due to the large particle size. However, the dispersion of KAUST-8@BPEI in n-butanol was obviously homogeneous, which could be attributed to the grafting of BPEI improving interface compatibility.

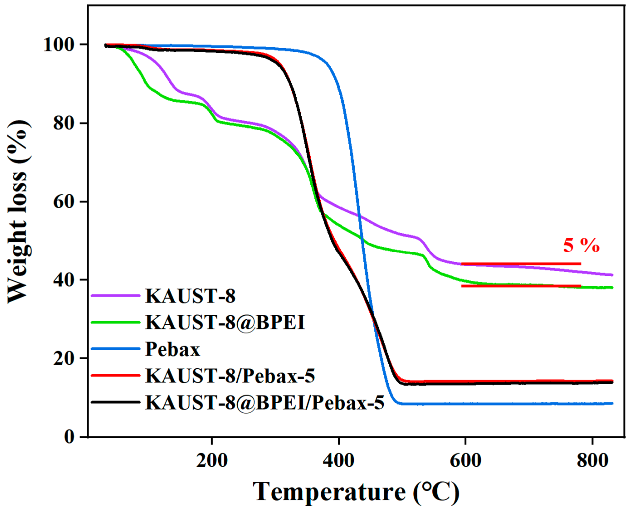

Figure S2. TGA for KAUST-8, KAUST-8@BPEI, neat membrane, KAUST-8/Pebax-5, and KAUST-8@BPEI/Pebax-5.

 https://doi.org/10.3390/membranes15010026

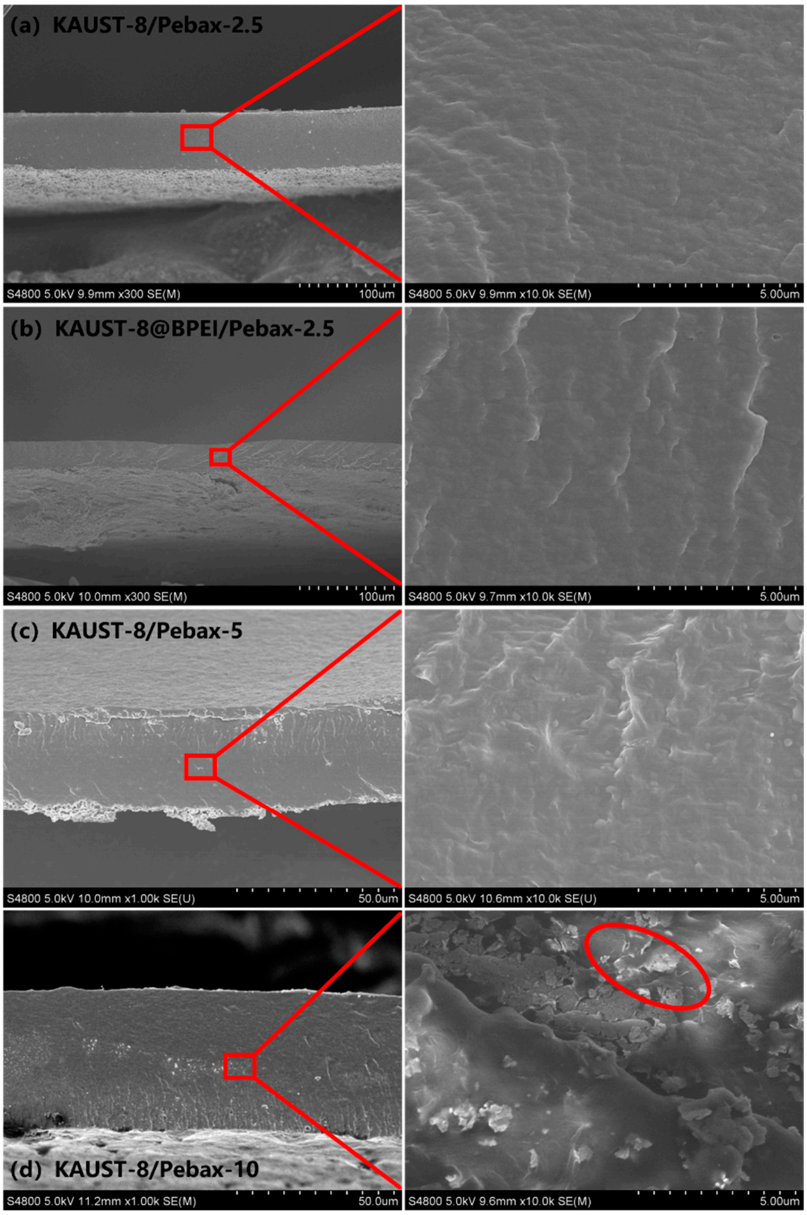

Figure S3. Cross-sectional SEM images for (**a**) KAUST-8/Pebax-2.5, (**b**) KAUST-8@BPEI/Pebax-2.5, (**c**) KAUST-8/Pebax-5, and (**d**) KAUST-8/Pebax-10. Left: low magnification, Right: high magnification, filler agglomeration is highlighted in the red circle in the inset in Figure S3d.

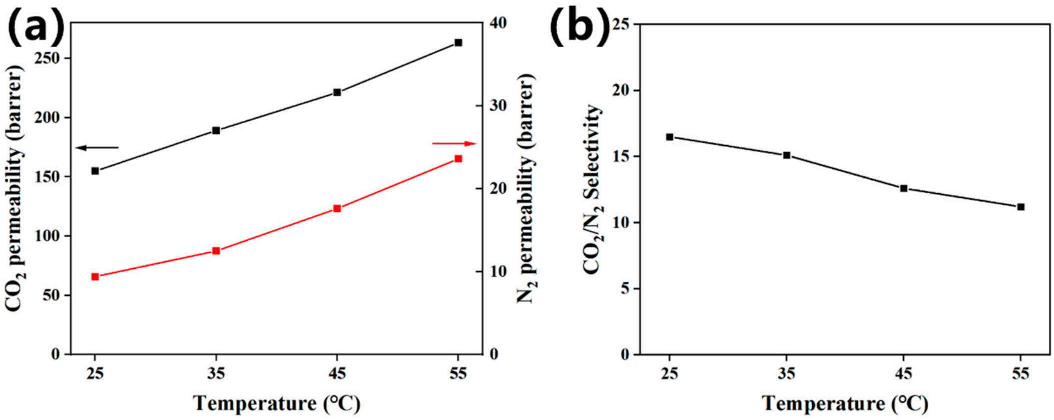

Figure S4. Temperature effect on the change of (**a**) CO_2 and N_2 permeability and (**b**) CO_2/N_2 selectivity of KAUST-8@BPEI-5/Pebax.

Table S1. Membrane thickness effect on membrane separation performance of CO_2 and N_2 permeability and CO_2/N_2 selectivity.

Membrane thickness (μm)	Gas permeability		Gas selectivity
	CO_2 (Barrer)	N_2 (Barrer)	CO_2/N_2
52.3	156.5	9.7	16.1
39.1	162.4	10.3	15.8
59.9	147.3	9.9	14.9
54.6	157.2	10.1	15.6
69.3	143.4	9.6	14.9
44.9	153.1	9.7	15.8

Notice: Representative MMMs (KAUST-8@BPEI/Pebax-5) were chosen for tests. The test condition: 1 bar and 25 ° C for single gas.

With different thickness, the CO_2 permeability, N_2 permeability and CO_2/N_2 selectivity of MMMs ranged from 143.4 to 162.4 Barrer, 9.6 to 10.3 Barrer and 14.9 to 16.1, separately, which were considered as normal floating ranges. It can be concluded that membrane thickness has a slight effect on membrane separation performance and the results are credible.

Table S2. Flow rates effect on membrane separation performance of CO_2 and N_2 permeability and CO_2/N_2 selectivity.

feed gas/sweep gas (mL/min)	Gas permeability		Gas selectivity
	CO_2 (Barrer)	N_2 (Barrer)	CO_2/N_2
10/10	137.4	9.3	14.8
20/20	156.5	9.7	16.1
30/30	153.7	9.6	16
40/40	158.9	/	/

Notice: Representative MMMs (KAUST-8@BPEI/Pebax-5) were chosen for tests. The test condition: 1 bar and 25 ° C for single gas. "/" means that the N_2 concentration is too low to be detected by the gas chromatography and thus the N_2 permeability and CO_2/N_2 selectivity can't be calculated.

As the flow rate raised from 10 to 20 mL/min, both the gas permeability and selectivity increased, indicating the phenomenon of concentration polarization caused by the low flow rate [1]. When the flow rate increased to 30 mL/min, the gas permeability and selectivity were almost unchanged. When the flow rate was further increased to 40 mL/min, the N_2 concentration of the mixed gas sent into the gas chromatography was too low to be detected. To sum up, the flow rate of 20 mL/min is the optimal experimental condition.

Table S3. BET surface area and pore volume of KAUST-8 and KAUST-8@BPEI.

MOF	BET surface area(m^2/g)	pore volume(cm^3/g)
KAUST-8	489.9	0.1963
KAUST-8@BPEI	433.1	0.1814

Table S4. The atomic concentration of Al, C, N, and Ni for KAUST-8 and KAUST-8@BPEI from XPS, respectively.

Num	Name	Binding energy (eV)	Atomic concentration (%)
KAUST-8	Al 2p	71.8	25.3
	C 1s	283.2	56.0
	N 1s	397.6	12.3
	Ni 2p	853.9	6.5
KAUST-8@BPEI	Al 2p	71.8	26.1
	C 1s	283.1	55.4
	N 1s	397.5	12.5
	Ni 2p	853.5	5.9

Table S5. Membrane separation performance comparison between this work and other published works.

Polymer	Filler	Filler loading (%)	Test Conditions	CO_2 permeability (Barrer)	Enhancement ratio (%)	CO_2/N_2 selectivity	Enhancement ratio (%)	Ref.
Pebax 1074	SAPO-23	5	30 °C, 1.5 bar	98.2	−32.4	72	275	[2]
Pebax 2533	PGO	0.02	35 °C, 1 bar	380.44	4.34	24.19	1.64	[3]
Pebax 1074	ZnO	8	25 °C, 3 bar	152.27	37.6	62.15	24.1	[4]
Pebax 1074/PEG-200	MgO	8	25 °C, 2 bar	210.1	225	60.9	24	[5]
XLPEO	KAUST-8	30	25 °C, 2 bar	744	277.7	81.7	28.7	[6]
Pebax 1657	ZIF-8	5	20 °C, 1 bar	99.7	25	59.6	25	[7]
Pebax 1657	NH_2-MIL-53	10	35 °C, 10 bar	149.1	174.1	55.5	39.4	[8]
Pebax 1657	Fumed silica	10	25 °C, 12 bar	72.91	45.9	113.92	−11.1	[9]
Pebax 1657	CMC-TiO_2	3	25 °C, 20 bar	194.6	60	82.4	30	[10]
Pebax 1657	CNs-600	0.5	25 °C, 4 bar	100.87	14.5	77.54	39.5	[11]
Pebax 1657	MXene	1	30 °C, 2 bar	148	66.3	63	18.9	[12]
Pebax 1657	SiO_2	1	25 °C, 4 bar	73.65	44	81.82	33	[13]
Pebax 1657	NaX	2	25 °C, 4 bar	50.7	−0.9	107.13	74.1	[13]
Pebax 2533	SUM-9	1	35 °C, 6 bar	539	130.3	24.69	11.2	[14]
Pebax 1074	KAUST-8	5	25 °C, 1 bar	86.9	22	13	9.2	This work
Pebax 1074	KAUST-8@BPEI	5	25 °C, 1 bar	156.5	119.8	16.1	35.3	This work

References

1. Chen, H.; Zhou, Y.; Sun, J.; Liu, Y.; Zhong, Y.; Du, W.; Lan, J. An Experimental Study of Membranes for Capturing Water Vapor from Flue Gas. *J. Energy. Inst.* **2018**, *91*, 339–348, doi:10.1016/j.joei.2017.02.007.
2. Zhang, S.; Zheng, Y.; Wu, Y.; Zhang, B. Fabrication of Pebax/SAPO Mixed Matrix Membranes for CO_2/N_2 Separation. *J. Appl. Polym. Sci.* **2021**, *138*, 51336, doi:10.1002/app.51336.
3. Casadei, R.; Giacinti Baschetti, M.; Yoo, M.J.; Park, H.B.; Giorgini, L. Pebax®® 2533/Graphene Oxide Nanocomposite Membranes for Carbon Capture. *Membranes* **2020**, *10*, 188, doi:10.3390/membranes10080188.
4. Azizi, N.; Mohammadi, T.; Behbahani, R.M. Synthesis of a PEBAX-1074/ZnO Nanocomposite Membrane with Improved CO_2 Separation Performance. *J. Energy. Chem.* **2017**, *26*, 454–465, doi:10.1016/j.jechem.2016.11.018.
5. Azizi, N.; Jahanmahin, O.; Homayoon, R.; Khajouei, M. A New Ternary Mixed-Matrix Membrane (PEBAX/PEG/MgO) to Enhance CO_2/CH_4 and CO_2/N_2 Separation Efficiency. *Korean J. Chem. Eng.* **2023**, *40*, 1457–1473, doi:10.1007/s11814-023-1391-5.
6. Hou, R.; Wang, S.; Wang, L.; Li, C.; Wang, H.; Xu, Y.; Wang, C.; Pan, Y.; Xing, W. Enhanced CO_2 Separation Performance by Incorporating KAUST-8 Nanosheets into Crosslinked Poly(Ethylene Oxide) Membrane. *Sep. Purif. Technol.* **2023**, *309*, 123057, doi:10.1016/j.seppur.2022.123057.
7. Zheng, W.; Ding, R.; Yang, K.; Dai, Y.; Yan, X.; He, G. ZIF-8 Nanoparticles with Tunable Size for Enhanced CO_2 Capture of Pebax Based MMMs. *Sep. Purif. Technol.* **2019**, *214*, 111–119, doi:10.1016/j.seppur.2018.04.010.
8. Meshkat, S.; Kaliaguine, S.; Rodrigue, D. Mixed Matrix Membranes Based on Amine and Non-Amine MIL-53(Al) in Pebax® MH-1657 for CO_2 Separation. *Sep. Purif. Technol.* **2018**, *200*, 177–190, doi:10.1016/j.seppur.2018.02.038.
9. Aghaei, Z.; Naji, L.; Hadadi Asl, V.; Khanbabaei, G.; Dezhagah, F. The Influence of Fumed Silica Content and Particle Size in Poly (Amide 6-b-Ethylene Oxide) Mixed Matrix Membranes for Gas Separation. *Sep. Purif. Technol.* **2018**, *199*, 47–56, doi:10.1016/j.seppur.2018.01.035.
10. Shamsabadi, A.A.; Seidi, F.; Salehi, E.; Nozari, M.; Rahimpour, A.; Soroush, M. Efficient CO_2-Removal Using Novel Mixed-Matrix Membranes with Modified TiO_2 Nanoparticles. *J. Mater. Chem. A* **2017**, *5*, 4011–4025, doi:10.1039/C6TA09990D.
11. Wang, H.; Zheng, W.; Yang, X.; Ning, M.; Li, X.; Xi, Y.; Yan, X.; Zhang, X.; Dai, Y.; Liu, H.; et al. Pebax-Based Mixed Matrix Membranes Derived from Microporous Carbon Nanospheres for Permeable and Selective CO_2 Separation. *Sep. Purif. Technol.* **2021**, *274*, 119015, doi:10.1016/j.seppur.2021.119015.
12. Shi, F.; Sun, J.; Wang, J.; Liu, M.; Yan, Z.; Zhu, B.; Li, Y.; Cao, X. MXene versus Graphene Oxide: Investigation on the Effects of 2D Nanosheets in Mixed Matrix Membranes for CO_2 Separation. *J. Membr. Sci.* **2021**, *620*, 118850, doi:10.1016/j.memsci.2020.118850.
13. Salehi Maleh, M.; Raisi, A. Comparison of Porous and Nonporous Filler Effect on Performance of Poly (Ether-Block-Amide) Mixed Matrix Membranes for Gas Separation Applications. *Chem. Eng. Res. Des.* **2019**, *147*, 545–560, doi:10.1016/j.cherd.2019.05.038.
14. Feng, X.; Qin, Z.; Lai, Q.; Zhang, Z.; Shao, Z.-W.; Tang, W.; Wu, W.; Dai, Z.; Liu, C. Mixed-Matrix Membranes Based on Novel Hydroxamate Metal-Organic Frameworks with Two-Dimensional Layers for CO_2/N_2 Separation. *Sep. Purif. Technol.* **2023**, *305*, 122476, doi:10.1016/j.seppur.2022.122476.
